# A Multi-Level Nonlinear Cumulative Fatigue Damage Life Prediction Model Considering Load Loading Effects

**DOI:** 10.3390/ma18173973

**Published:** 2025-08-25

**Authors:** Bowen Yang, Junzhou Huo

**Affiliations:** 1 School of Mechanical Engineering, Shenyang University of Technology, Shenyang 110870, China; 2 School of Mechanical Engineering, Dalian University of Technology, Dalian 116024, China

**Keywords:** load ratio, multi-level loading, Manson-Halford model, fatigue damage

## Abstract

Fatigue damage failure is a process where the mechanical properties of different materials continuously degrade under the action of cyclic loads. The cumulative analysis of fatigue damage has a significant impact on the service structure of major equipment. This paper starts from the mechanism of fatigue damage evolution, comprehensively considers the influence of the order of high-low cycle load mixed cyclic loading on the fatigue life performance, and based on the Manson-Halford nonlinear fatigue damage accumulation theory and the mechanism of relative cumulative damage, a new nonlinear damage accumulation fatigue life model is established, and a fatigue damage accumulation influencing factor *D_cr_* is introduced to improve the prediction accuracy of the model. The new model proposed in this paper is verified through multi-level fatigue load data. By comparing the prediction results with other models under the same experimental conditions, the fatigue life prediction error precision of the new model is the best in similar cases, generally with an error precision between 10% and 20%, which proves the effectiveness and accuracy of the nonlinear damage accumulation model proposed in this paper. At the same time, the improved method in this paper has better stability while ensuring prediction accuracy.

## 1. Introduction

**Figure 1:**
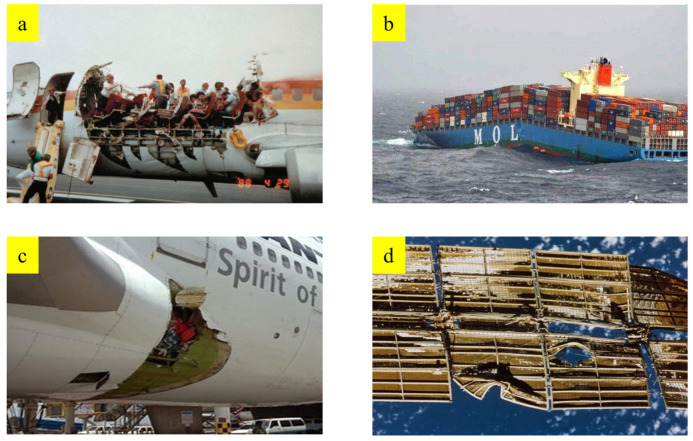
Fatigue Damage Failure and Fracture in Major Equipment. (**a**) Aircraft fuselage fracture (**b**) brittle fracture in a welded ship (**c**) Aircraft cabin skin peeling off (**d**) Deformation and cracking of spacecraft.

The fatigue behavior of metal materials is crucial. It has important applications in the main load-bearing structures of major equipment, including aircraft components, shield machine cutterheads, automotive parts, and other structures. These parts or structures will continue to experience fatigue load conditions. The mechanical properties of the components will continue to accumulate and fail during actual loading. Therefore, accurate prediction of fatigue life is crucial for the design optimization process. Fatigue failure is one of the most common modes of failure in major equipment, posing a significant threat to the safe operation of equipment, including aircraft, trains, ships, bridges, oil platforms, as well as aircraft engine turbine blades, steam turbine blades, etc., as shown in Figure 1. Due to the complex structure and complex loads of major equipment, the main load-bearing structures typically undergo extremely complex loads during operation, and are also subject to the effects of environmental corrosion. As a result, cracks tend to initiate at stress concentration areas or structurally weaker locations, and microcracks slowly grow under the action of service loads until they form macroscopic cracks, which greatly threaten the safety of the structure. Incidents caused by fatigue failure of similar major equipment still occur from time to time, making people increasingly concerned about structural fatigue issues. In fact, more fatigue does not lead to accidents, as fatigue failure is cumulative and generally requires a certain operating time to occur. Moreover, the location of the failure is uncertain before it occurs, and there are no obvious signs, so fatigue failure is hidden and very dangerous, which must be given high attention. To ensure the safe operation of equipment, it is necessary to conduct real-time detection and timely maintenance of the fatigue of the structure.

Fatigue is an accumulation process of damage where the material properties continuously degrade under the action of cyclic loads. The analysis of damage accumulation plays a crucial role in preventing fatigue failure, and the mechanism of damage evolution is one of the important issues in the study of fatigue behavior. Continuous loading under cyclic loading leads to cumulative fatigue damage in structural materials [1]. Local damage caused by cyclic loads is an accumulation process composed of crack initiation, propagation, and fracture. During the cyclic loading process, local plastic deformation occurs in the area with the highest stress, leading to crack initiation. As the number of loading cycles that the part undergoes continues to accumulate, the propagation of cracks also increases. When the load reaches a certain number of cycles, the cracks will lead to structural failure and fracture. It is necessary to monitor the load history in real-time to predict the fatigue life of equipment, thereby achieving a fatigue load assessment of in-service equipment [2]. It is crucial to effectively and timely predict and evaluate the stress, strain, external impact, and fatigue damage of structures [3]. The remaining fatigue life is calculated through future load prediction models and physical failure models. For fatigue monitoring of mechanical structures, it is usually based on cumulative damage theory to calculate the fatigue life. However, a large number of experimental studies and practical engineering applications have shown that fatigue life prediction based on Miner’s linear damage theory may have non-conservative results. Since then, a large number of damage models have been proposed [4,5,6].

Since the loading sequence effect is an important factor affecting the fatigue damage accumulation rule, Jang et al. [7] considered the effect of loading sequence and introduced specific influencing parameters into the fatigue damage accumulation model. Qin [8] and Xie [9] et al. introduced the concept of damage curves based on the fracture expansion concept theory, considering the amount of damage at each load level as a power function related to the load. Xia [10] summarized the fatigue damage assessment methods under random loads and proposed a fatigue cumulative damage model based on the equal damage curve. Bjørheim [11] et al. used the damage stress function of fatigue life to establish a nonlinear model for assessing cumulative fatigue damage under multi-level loading. Jiang [12] et al. proposed a low-cycle fatigue model considering the interaction of loads to predict the fatigue life under random loads. Mohamed et al. [13] considered the damage accumulation process based on the influence of material fatigue performance, considering the correlation between load paths. Lin et al. [14] proposed a fatigue life model considering the evolution of structural fatigue damage through failure mode analysis of residual strength. Gao et al. [15] proposed a nonlinear model considering the influence of load sequence by introducing load-related parameters. Zhou et al. [16] proposed a Corten-Dolan cumulative damage model considering wear degree. Yuan et al. [17] and others, based on the DCA model, established a fatigue damage model considering residual strength degradation caused by variable amplitude cycles under stress amplitude. Due to the importance and complexity of key structures in aerospace, there is still a lack of a complete and effective monitoring system for real-time prediction of the fatigue life of key structures [18]. Intelligent node technology is used to monitor the damage state, external impact load, and usage environment of key parts of the structure in real-time, obtaining the fatigue load spectrum during use. Based on big data algorithms, the dominant load spectrum is predicted, and the remaining fatigue life is calculated. Ensure to reduce manufacturing costs and extend the service life of the aircraft. Predict fatigue life failure and provide timely maintenance warning for flight shutdown. Therefore, the intelligent node monitoring technology studied in this paper can obtain the damage state and evolution law of key structures in the service process in real-time. Timely warning provides important support for flight shutdown maintenance and service life prediction.

In summary, due to the complexity of the structure of major equipment, the complexity of the load, the complexity of manufacturing, and the complexity of the service environment, the law of force flow transfer in the main load-bearing structure of major equipment is complex, the structural weak position is difficult to locate, the structure is difficult to detect, and the whole life cycle is difficult to assess, which is prone to cause catastrophic accidents and poses a huge threat to the economy and people’s life safety. This article starts from the evolution mechanism of fatigue damage and comprehensively considers the influence of mixed cyclic loading sequence of high and low cycle loads on fatigue life performance. Based on the theory of nonlinear fatigue damage accumulation, a new nonlinear damage accumulation fatigue life model is established, and the influence factors of fatigue damage accumulation are introduced to improve the prediction accuracy of the model. Therefore, accurate fatigue life analysis, especially the life assessment of large and complex equipment in fields such as aerospace, rail transit, and ships, has important practical significance and engineering significance, and has become a bottleneck that needs to be urgently solved for the healthy and efficient operation of major equipment.

## 2. Theoretical Methods

The damage curve method is one of the most classic nonlinear cumulative damage theories, which has been optimized and improved by scholars, The model proposed by Manson-Halford [19] was widely used in later research, and the calculation formula for the model is:

(1) D=(nNf)(NfNRef)

Among them, *D* is the amount of damage, *n* is the number of cycles, *N_f_* is the fatigue life of load, *N_Ref_* is the damage life.

The damage of the material under the *i* level load cycle is:

(2) $$\left\{\begin{array}{c} D=\sum_{i=1}^{n}(\frac{n_{i}}{N_{fi}}{)}^{a_{i}}\\ a_{i}=BN_{fi}^{\beta } \end{array}\right.$$

In the formula, *n_i_* is the *i* level cyclic load, *N_fi_* is the *i* level fatigue life, *a_i_* is the fitting parameter, *β* is a constant related to material properties. Similar to Miner’s law, when the critical damage value is 1, the material structure fails. After preliminary experiments and experience accumulation, the empirical constant of 0.4 is generally directly taken for *a_i_* in practical applications.

Under the two-stage variable amplitude load spectrum, The Manson Halford model formula is:

(3) $$(\frac{n_{1}}{N_{f1}}{)}^{(\frac{N_{f1}}{N_{f2}}{)}_{0.4}}+\frac{n_{f2}}{N_{f2}}=1$$

In the formula, *n*_1_ is the 1 level cyclic load, *N_f_*_1_ is the 1 level fatigue life, *n*_2_ is the 2 level cyclic load, *N_f_*_2_ is the 2 level fatigue life.

**Figure 2:**
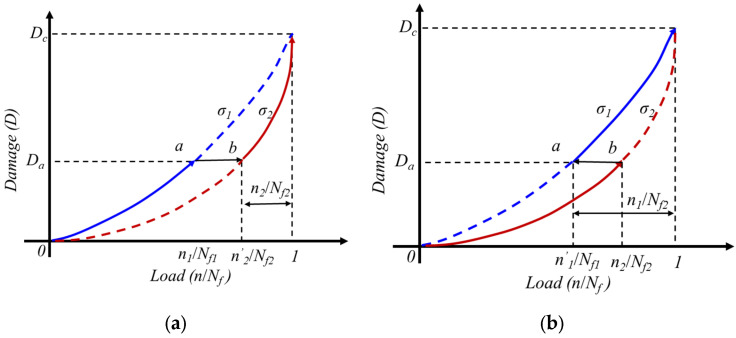
Damage Accumulation Curve Considering Load Sequence (**a**) High and Low Loading (**b**) Low and High Loading [20].

Due to the Manson-Halford theory of the damage curve method considering the order of load action and stress magnitude, it is more accurate to calculate the damage of two-stage loads. However, when extended to multi-level loads, due to the complex coupling effect between loads, there are also errors in the calculation results. Taking the cumulative damage theory diagram under secondary loading as an example. The cumulative processes of fatigue damage under high and low loads are shown in Figure 2, respectively. The accumulation of fatigue damage is related to stress levels, and the higher the load level, the faster the rate of damage accumulation. a_1_ and a_2_ respectively represent different stress levels, and D_a_ and D_b_ respectively represent the cumulative fatigue damage at the corresponding stress levels of a_1_ and a_2_. Considering the influence of loading sequence on fatigue damage, the cumulative process of fatigue damage under variable amplitude load is shown in Figure 2, with load level *σ*_1_ > *σ*_2_. When the order of load action is high low, the damage accumulation process is: o-a-b; When the load sequence is low high, the damage accumulation process is o-b-a. It can be observed that when the damage accumulates to the same level, the number of cycles with high low load sequence is less than the number of cycles with high low load sequence.

Under the loading of the first stage load, the number of cycles is *n*_1_, and under the continuous loading of the second stage load *n*_2_, it ultimately leads to fatigue failure of the material structure, with a number of cycles of *N_f_*_2_. The fatigue life of the first level load is *N_f_*_1_, and the fatigue life of the second level load is *N_f_*_2_.

Since the fatigue damage values of *a* and *b* are the same at the load level, the relationship between the load cycle and the equivalent cycle ratio is:

(4) $$(\frac{n_{1}}{N_{f1}})={\frac{n_{2}}{N_{f2}}}^{(\frac{N_{f1}}{N_{f2}}{)}^{0.4}}$$

In the formula, the number of cycles at the *N_f_*_2_ life level is *n*_2_, and the equivalent damage corresponds to the initial cycle ratio.

The fatigue life problem of high and low cycle composite loading is one of the common fatigue failure modes under the coupling of high cycle stress and low cycle stress [20]. Under high and low cycle composite fatigue loading, high stress levels under low cycle cyclic loading cause severe damage to the material; The stress level under high cycle loading is low, but the high frequency also has a serious impact on the material. There is currently a lack of sufficient and accurate research on the complex interaction coupling damage caused by high-low cycle composite loading. The current experiment lacks complete performance data and cannot predict the fatigue life of high and low cycle composite loading well. Therefore, in order to study the actual damage process and fatigue life of key structures during service, it is necessary to conduct in-depth research on them.

**Figure 3:**
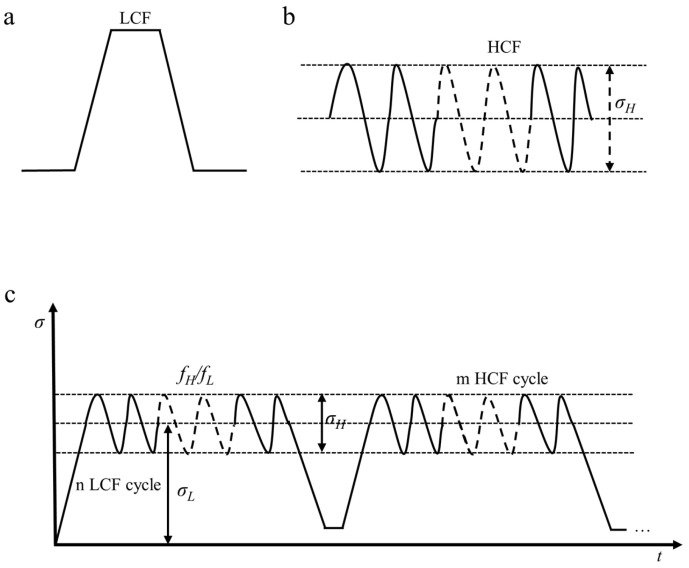
High low cycle composite fatigue load spectrum (**a**) Low cycle load spectrum (**b**) High cycle load spectrum (**c**) High low cycle composite load spectrum.

The load spectrum waveforms of low cycle fatigue and high cycle fatigue for service structures subjected to complex alternating loads in actual engineering are shown in Figure 3. Low frequency and high amplitude low cycle cyclic loads (Figure 3a) and high frequency and low amplitude high cycle cyclic loads (Figure 3b) jointly composite and interact with key structures. However, in practical engineering, the load spectrum of service structures is a random load spectrum. To ensure that it can be studied under complex test conditions, it is necessary to convert it into a typical test load spectrum through the principle of equivalent damage. The specific load waveform is shown in Figure 3c. Physical parameters such as high cycle stress *σ_H_*, high cycle stress frequency *f_H_*, low cycle stress *σ_L_*, and low cycle stress frequency *f_L_* are commonly used for research. At the same time, it is closely related to parameters such as the ratio of high cycle stress frequency to low cycle stress frequency.

The impact of different number of cycles under each level of load on the accumulation of subsequent fatigue damage is also different. Therefore, the relationship between the ratio of cycle loading times to fatigue life (loading cycle ratio) and fatigue damage can be used to represent the impact of load action sequence on fatigue damage, as shown in Figure 3. It can be found that:

When the loading order is high low,

(5) $$\sum \frac{n_{i}}{N_{fi}}<1$$

When the loading order is low high,

(6) $$\sum \frac{n_{i}}{N_{fi}}>1$$

Based on the classic model mentioned above, this paper proposes a complex fatigue damage life prediction model for high and low cycle coupled loading, considering the influence of high and low cycle coupled loading and multi-level spectrum on material structure, which is more universal and accurate.

When the external stress applied by the actual service structure is lower than the yield strength *σ_z_* of the material’s inherent properties, there exists a relationship between the loading stress amplitude of the material and the fatigue life of the structure:

(7) $$\sigma_{a}=\sigma_{f}^{\prime }N_{f}^{b}$$

Among them, *σ_a_* is the stress amplitude of the material, $\sigma_{f}^{\prime }$ is the fatigue strength, *b* is the material parameter.

Due to the complex causes of structural fatigue, fatigue damage is a key factor affecting the lifespan. When the structure experiences complete failure, the use of damage models for calculation is not considered. The cumulative value of the ratio of cyclic load to load fatigue life is the fatigue damage value.

According to the equivalent theory of fatigue cumulative damage characteristics of materials, the expression for the cyclic ratio is:

(8) $$(\frac{n_{2}^{\prime }}{N_{f2}})={\frac{n_{1}}{N_{f1}}}^{(\frac{N_{f1}}{N_{f2}})\alpha }$$

In the formula, $n_{2}^{\prime }$ is the number of cycles required to increase the damage from Coordinate axis O along the damage curve *a* to *b* under stress *σ*_2_.

When the cumulative damage *D* reaches the critical value after *n*_2_ cycles of *σ*_2_, the material will experience fatigue failure, with a critical damage value of D = 1. At this point, the criterion for fatigue failure under the secondary load spectrum can be expressed as:

(9) $$\frac{n_{2}^{\prime }}{N_{f2}}+\frac{n_{2}}{N_{f2}}={\frac{n_{1}}{N_{f1}}}^{(\frac{N_{f1}}{N_{f2}})\alpha }+\frac{n_{2}}{N_{f2}}=1$$

Based on this, the expression for the three-level loading damage model is:

(10) $$\{(\frac{n_{1}}{N_{f1}}{)}^{(\frac{N_{f1}}{N_{f2}})\alpha }+\frac{n_{2}}{N_{f2}}{\}}^{(\frac{N_{f2}}{N_{f3}})\alpha }+\frac{n_{3}}{N_{f3}}=1$$

Due to the improved accuracy of the Manson Halford damage model’s three-level loading damage prediction compared to the Miner criterion, it cannot meet the requirements of practical engineering research. Therefore, this paper introduces a correction factor to improve and expand the multi-level loading damage model, as shown below:

(11) $$\left\{\begin{array}{c} \{[(\frac{n_{1}}{N_{f1}}{)}^{a_{1}}+\frac{n_{2}}{N_{f2}}{]}^{a_{2}}+\ldots +\frac{n_{i-1}}{N_{f(i-1)}}{\}}^{a_{i-1}}+\frac{n_{i}}{N_{fi}}=D_{cr}\\ a_{i-1}=\beta_{i-1}(\frac{N_{f\left(i-1\right)}}{N_{fi}}{)}^{0.4\beta_{i-1}}\;,\;\beta_{i-1}=\min\{\frac{\sigma_{i}}{\sigma_{i-1}},\frac{\sigma_{i-1}}{\sigma_{i}}\} \end{array}\right.$$

Among them, *n_i_* is the *i* level cyclic load, *N_fi_* is the *i* level fatigue life, $\beta_{i-1}$ is the cyclic stress ratio, *D_cr_* is the fatigue damage correction factor. *D_cr_* needs to be experimentally determined.

However, considering the influence of load loading sequence on fatigue life, the fatigue life is less than 1 under high low loading and greater than 1 under low high loading. Therefore, this paper introduces the fatigue damage correction factor *D_cr_* and improves and extends it to the multi-level loading damage model.

This article proposes a universal formula. Suitable for most metal materials and other isotropic similar materials. Mainly researching commonly used materials for major equipment. For example: steel, iron, aluminum, etc.

## 3. Fatigue Test

### 3.1. Test Material

**Figure 4:**
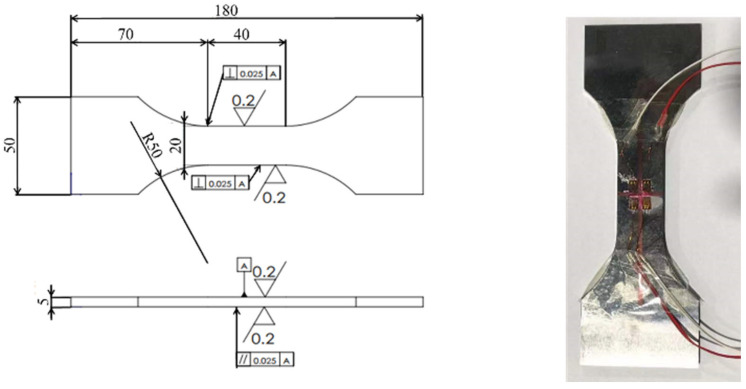
Dimensions of standard sample.

The experimental material is Q345D, which is a low-alloy high-strength structural steel. Q345D complies with ASTM A572/A572M-23, standards. Key weighing components commonly used in large and complex equipment [21]. Has good comprehensive performance and excellent fatigue resistance. Q345D was calibrated for mechanical performance testing based on the data results of the tensile test, as shown in Table 1. The sample material is Q345D. The tensile strength *σ*_s_ is 345 MPa. The surface roughness can reach Ra = 0.2 μm. The machining accuracy can reach 0.02 mm. The main structural dimensions of the design standard specimen are shown in Figure 4.

### 3.2. Test Equipment

**Figure 5:**
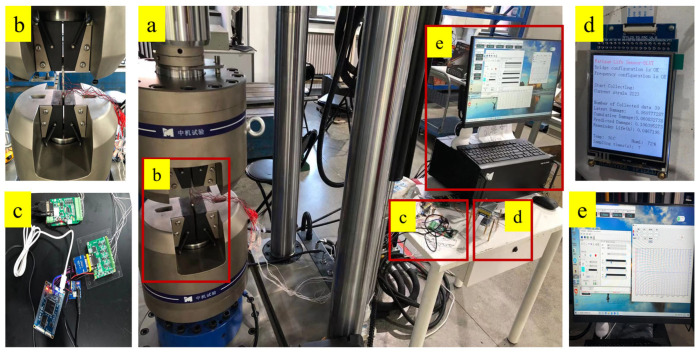
Fatigue life prediction test system. (**a**) Electro-hydraulic servo fatigue tensile testing machine, (**b**) Fatigue life prediction data display module, (**c**) Loading samples and strain monitoring, (**d**) Loading parameter and display data computer; (**e**) Life prediction acquisition and processing main module.

The standard sample fatigue test adopts a fatigue testing system designed by our team, as shown in Figure 5. The test bench adopts a 500 kN electro-hydraulic servo fatigue testing machine (type: SY197223F, location: Liaoning China), with a maximum testing frequency of 30 Hz. The equipment mainly includes the main unit, control unit, hydraulic unit, auxiliary unit, etc. Add the standard specimen to the fatigue testing machine and attach the strain gauge, and connect the self-made in-situ monitoring device. By uploading test data to the upper computer.

### 3.3. Fatigue Test

Metal fatigue testing requires material mechanics testing, fatigue material S-N curve testing, and multi-stage loading fatigue testing. Metal static mechanical tests can determine the basic mechanical parameters of Q345 material. The experiment is conducted under force control conditions, and the elastic modulus of the material is calculated by measuring the strain using strain gauges. During the experiment, the control method is force control. The applied load is a sine wave with a frequency of 10 Hz and a stress ratio of 0.1, with a cross-sectional area of 100 mm. Similarly, the force gradually increases in one cycle until the specimen deforms. Then the force gradually decreases until the sample stress returns to zero. Determine the maximum load based on mechanical test parameters and conduct two sets of tests at each point, for a total of ten sets of tests. In order to verify the accuracy of the multi-level fatigue life model, multiple sets of fatigue tests were conducted on the standard sample, and synchronized verification was carried out with other test data.

## 4. Results and Discussion

### 4.1. Static Test and Fatigue Test Results

**Figure 6:**
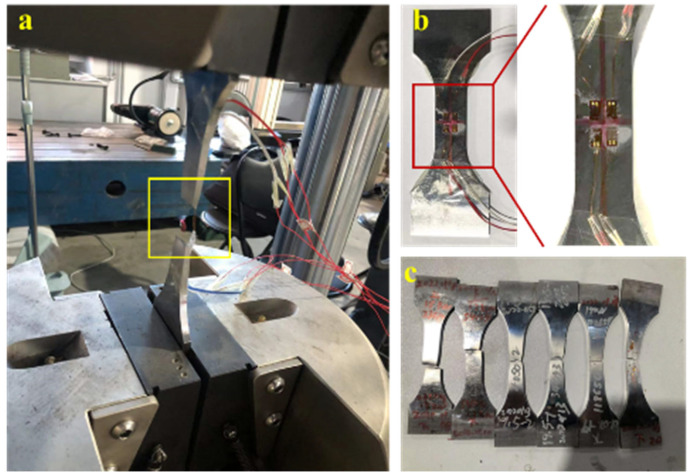
Fatigue test fracture of standard specimens. (**a**) Fracture of the loaded specimen in the fatigue test, (**b**) Real-time monitoring of dangerous locations by strain gauges, (**c**) A set of fatigue life fracture tests with different cyclic stresses.

In the fatigue test, the test specimen gradually accumulates damage under cyclic loading, as shown in Figure 6. The strain collection system monitors and evaluates the dangerous positions of the structure in real time and obtains the change patterns, and loads the test to the fracture state of the final sample. Load with different cyclic loads according to the design, and take the final fracture failure of the structure as the life cycle.

The basic mechanical parameters of the material in the model were determined through static testing. Based on the average of three sets of mechanical tests, the yield strength of Q345D material was 320 MPa and the tensile strength was 485 MPa. In engineering applications, longitudinal load-bearing steel bars in seismic structures must meet the mandatory requirement of a strength to yield ratio of ≥1.25 to avoid premature loss of bearing capacity. This article uses materials used in actual engineering experiments, resulting in a high strength to yield ratio.

According to the national standard requirements for fatigue tensile testing, at least 4 different load levels should be selected for laboratory level SN curves, and 2 valid data points should be selected for each group. Engineering problems require selecting at least 8 different load levels, with 6 valid data points per group. Therefore, the maximum load for each group in this experiment is determined to be 80%, 75%, 70%, 65%, and 60% of the tensile strength. The frequency is 10 Hz, the stress ratio is 0.1, and the effective area is 100 mm^2^. Finally, each group selects two valid experimental data for fitting. The specific experimental results are shown in Table 2.

**Figure 7:**
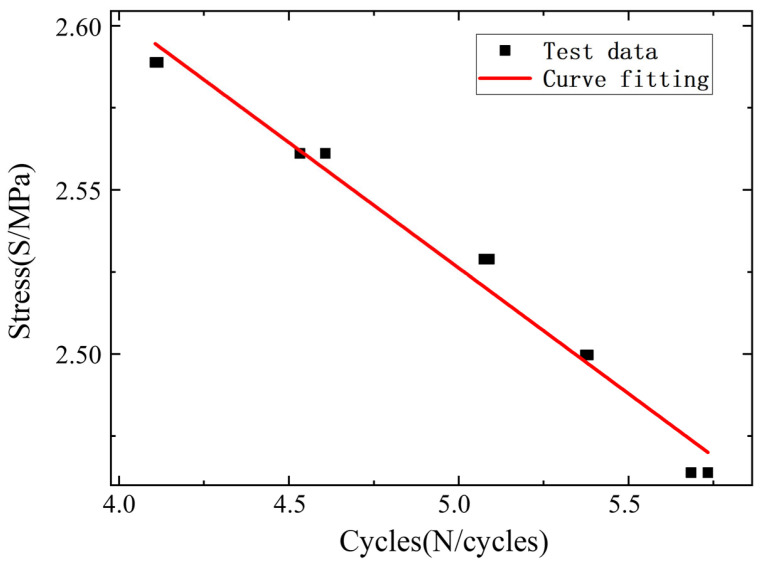
Fatigue life S-N prediction model.

If the test specimen does not fracture after exceeding 10^6^ cycles, it is concluded to have achieved infinite life. When the load is 13.3 kN in this experiment, it has exceeded 10 times without breaking, and it is considered that the infinite life range of the material has been reached. Fit the fitting curve of material Q345D based on ten sets of load spectrum data, as shown in Figure 7. The logarithmic fitting curve is taken for the horizontal and vertical axes.

### 4.2. Multi-Level Loading Fatigue Test Results

In order to verify the accuracy of the multi-stage fatigue life model considering load effects proposed in this article, three sets of experiments were conducted at each test point, and the average value was calculated. The fatigue life prediction under different multi-stage loads was verified based on single-stage loading experiments, and the effectiveness of the improved model proposed in this paper was validated. This article selects four classic models (Manson-Halford, YGao, Guo, Yang) for comparative analysis. The specific results are shown in Table 3, Table 4, Table 5 and Table 6. 

**Figure 8:**
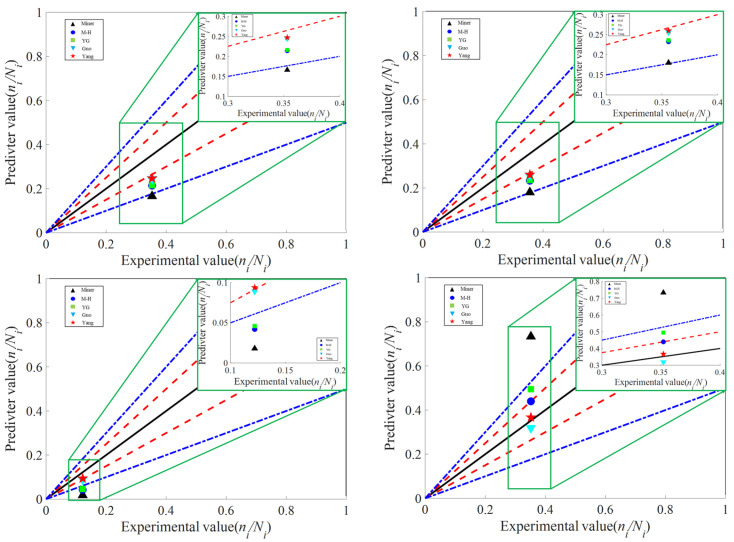
Fatigue verification experiment under different level loading.

Two sets of third level cyclic loads, one set of fourth level cyclic load, and one set of fifth level cyclic load are gradually applied with different reference data, and the fatigue limit life is obtained based on the S-N curve of single level loading. According to Figure 8, the black line represents a 1 fold dispersion band, the red line represents a 1.25 fold dispersion band, and the blue line represents a 1.5 fold dispersion band. The predicted results are compared with Miner’s A comparison was made between the M-H, YG, Yue, Guo models and the model proposed in this paper. The model proposed in this article was compared with four other models and found to have higher accuracy in different experimental groups, closer to the 1.25 fold dispersion band. It can be observed that when the loading mode is high low, the models mostly provide significantly non conservative prediction results, while when the loading mode is low high, the models mostly provide overly conservative prediction results. Verified that the improved model proposed in this article has the highest accuracy under the same load parameters.

Therefore, this article compares the predicted results with Miner M-H, YG, and Guo models. Compare and verify within a limited cycle life range (<10^−6^). Verified the accuracy of the Yang model. Under various levels of cyclic loading, the method proposed in this paper calculates a relative error smaller than other models under the same conditions. The accuracy of the first group’s three-level load forecasting is 30.09%, the accuracy of the second group’s three-level load forecasting is 26.07%, the accuracy of the fourth level load forecasting is 23.01%, and the accuracy of the fifth level load forecasting is 4.25%. Compared with other methods, the nonlinear damage accumulation model proposed in this paper has the smallest error. The error comparison analysis of multi-level different models, as shown in Table 7, is significantly better than Miner’s linear damage accumulation theory Nonlinear damage accumulation models such as M-H, YG, Guo, etc.

## 5. Conclusions

This article studies the problem of predicting the fatigue life of key structures in service equipment under multi-level loading. By analyzing the theory of cumulative fatigue damage calculation, it addresses the difficulty of predicting fatigue life under high and low cyclic mixed loads during actual equipment use.

(1) A multilevel load fatigue life pre-diction model considering load effects is proposed based on the Manson Harvard theory, which introduces the cumulative factor of fatigue damage.

(2) The accuracy of the model was verified through multiple sets of experimental reference data. The errors for level two to five are: 30.09%, 26.07%, 23.01%, 4.25%. Under various levels of cyclic loading, the method proposed in this paper calculates a relative error smaller than other models under the same conditions.

(3) The improved fatigue life model proposed in this article has the best predictive performance among similar models. By combining practical engineering loads with multi-level loading changes, the long-term stable monitoring and fatigue life prediction of key structures in service equipment have been achieved, providing a theoretical basis while avoiding unexpected catastrophic accidents.

## Figures and Tables

**Table 1:** Mechanical properties of Q345D.

Material	$\mathrm{Strength}\sigma_{s}$/MPa	Density g/cm^3^	Elastic Modulus E/GPa	Poisson’s Ratio μ
Q345D	345	7.65	210	0.3

**Table 2:** Material S-N curve test.

Test	$\mathrm{Stress}\;\mathrm{Mean}\;\sigma_{m}$/MPa	Load F/kN	Cycles/Cycles
T1_1	388	19.4	12,772
T1_2	388	19.4	13,052
T2_1	364	18.2	40,502
T2_2	364	18.2	34,093
T3_1	338	16.9	123,135
T3_2	338	16.9	118,650
T4_1	316	15.8	240,707
T4_2	316	15.8	236,249
T5_1	291	14.6	483,611
T5_2	291	14.6	541,691

**Table 3:** Fatigue verification experiment under three-level loading.

Stress *σ_i_*	Cycle *n_i_*	Damage *n_i_*/*N_fi_*	Miner *n_i_/N_fi_*	M-H *n_i_/N_fi_*	YG *n_i_/N_fi_*	Guo *n_i_/N_fi_*	Yang *n_i_/N_fi_*
38	400,000	0.371	0.371	0.371	0.371	0.371	0.371
40	400,000	0.463	0.629	0.662	0.663	0.680	0.680
42	247,500	0.353	0.166	0.214	0.216	0.244	0.247

**Table 4:** Fatigue verification experiment under three-level loading.

Stress *σ_i_*	Cycle *n_i_*	Damage *n_i_*/*N_fi_*	Miner *n_i_*/*N_fi_*	M-H *n_i_*/*N_fi_*	YG *n_i_*/*N_fi_*	Guo *n_i_*/*N_fi_*	Yang *n_i_*/*N_fi_*
23.3	129,700	0.136	0.136	0.136	0.136	0.136	0.136
25.1	443,500	0.684	0.864	0.903	0.905	0.919	0.921
26	192,400	0.356	0.180	0.233	0.236	0.256	0.2601

**Table 5:** Fatigue verification experiment under five-level loading.

Stress *σ_i_*	Cycle *n_i_*	Damage *n_i_*/*N_fi_*	Miner *n_i_*/*N_fi_*	M-H *n_i_*/*N_fi_*	YG *n_i_*/*N_fi_*	Guo *n_i_*/*N_fi_*	Yang *n_i_*/*N_fi_*
28.95	400,000	0.273	0.273	0.273	0.273	0.273	0.273
30.48	400,000	0.336	0.727	0.756	0.756	0.77	0.775
32	400,000	0.409	0.391	0.445	0.448	0.480	0.483
35.05	82,400	0.122	−0.018	0.042	0.046	0.089	0.094

**Table 6:** Fatigue verification experiment under five-level loading.

Stress *σ_i_*	Cycle *n_i_*	Damage *n_i_*/*N_fi_*	Miner *n_i_*/*N_fi_*	M-H *n_i_*/*N_fi_*	YG *n_i_*/*N_fi_*	Guo *n_i_*/*N_fi_*	Yang *n_i_*/*N_fi_*
350	44	0.0008	0.0008	0.0008	0.0008	0.0008	0.0008
332	352	0.0048	0.9992	0.9982	0.9983	0.9976	0.9977
298	6160	0.0474	0.9944	0.9817	0.9835	0.9705	0.9731
254	59,840	0.2137	0.9470	0.8651	0.8796	0.7997	0.8183
201	440,000	0.352	0.7333	0.4397	0.4948	0.3186	0.3670

**Table 7:** Fatigue life error result analysis.

Level	Miner *n_i_*/*N_fi_*	M-H *n_i_*/*N_fi_*	YG *n_i_*/*N_fi_*	Guo *n_i_*/*N_fi_*	Yang *n_i_*/*N_fi_*
3-1	52.97%	39.41%	38.67%	30.88%	30.09%
3-2	49.43%	34.57%	33.62%	27.65%	26.07%
4-1	114.75%	65.58%	62.40%	26.98%	23.01%
5-1	108.32%	24.90%	40.57%	9.49%	4.25%

## Data Availability

The original contributions presented in this study are included in the article. Further inquiries can be directed to the corresponding author.
